# Supplementary material for: Flexible Graphite/PPG Hybrid Composite-Based Resistive Sensor for Sensing Organic Compounds
Source: Sensors (Basel). 2020 May 6;20(9):2651. doi: 10.3390/s20092651 (PMC7249171; doi:10.3390/s20092651)
Supplement: Supplementary file 1 [file sensors-20-02651-s001.zip › sensors-761390-supplementary1.pdf]

Supplementary Information for:

Flexible Graphite/PPG Hybrid Composite-Based Resistive Sensor for Sensing Organic Compounds

Do Hun Kim [1,†], Yang Soo Lee [2,†], Won Kyu Park [3,†], Jin Sun Yoo [1], Changup Shim [4], Young Joon Hong [5], Bong Kyun Kang [1], Dae Ho Yoon [2,*] and Woo Seok Yang [1,*]

[1] Nano Materials and Components Research Center, Korea Electronics Technology Institute, 25, Saenari-ro, Bundang-gu, Seongnam-si, Gyeonggi-do 13509, Korea; amazingcom@keti.re.kr (D.H.K.); yjs0415@keti.re.kr (J.S.Y.); kangbk84@keti.re.kr (B.K.K)

[2] School of Advanced Materials Science and Engineering, Sungkyunkwan University, 2066, Seobu-ro, Jangan-gu, Suwon-si, Gyeonggi-do 16419, Korea; koami@skku.edu

[3] Nano Material Division, Cheorwon Plasma Research Institute, Cheorwon, Gangwon-do 24047, Korea; wkpark@cpri.re.kr

[4] Division of Advanced Materials Engineering, Kongju National University, 1223-24, Cheonan-daero, Seobuk-gu, Cheonan-si, Chungcheongnam-do 31080, Korea; uhbi@naver.com

[5] Department of Nanotechnology and Advanced Materials Engineering, Sejong University, Seoul 05006, Korea; yjhong@sejong.ac.kr

* Correspondence: dhyoon@skku.edu (D.H.Y.); wsyang@keti.re.kr (W.S.Y.); Tel.: +82-31-290-7361 (D.H.Y.); +82-31-789-7256 (W.S.Y.)

† D.H. Kim, Y.S. Lee, and W.K. Park contributed equally to this work.

Received: 17 March 2020; Accepted: 29 April 2020; Published: date

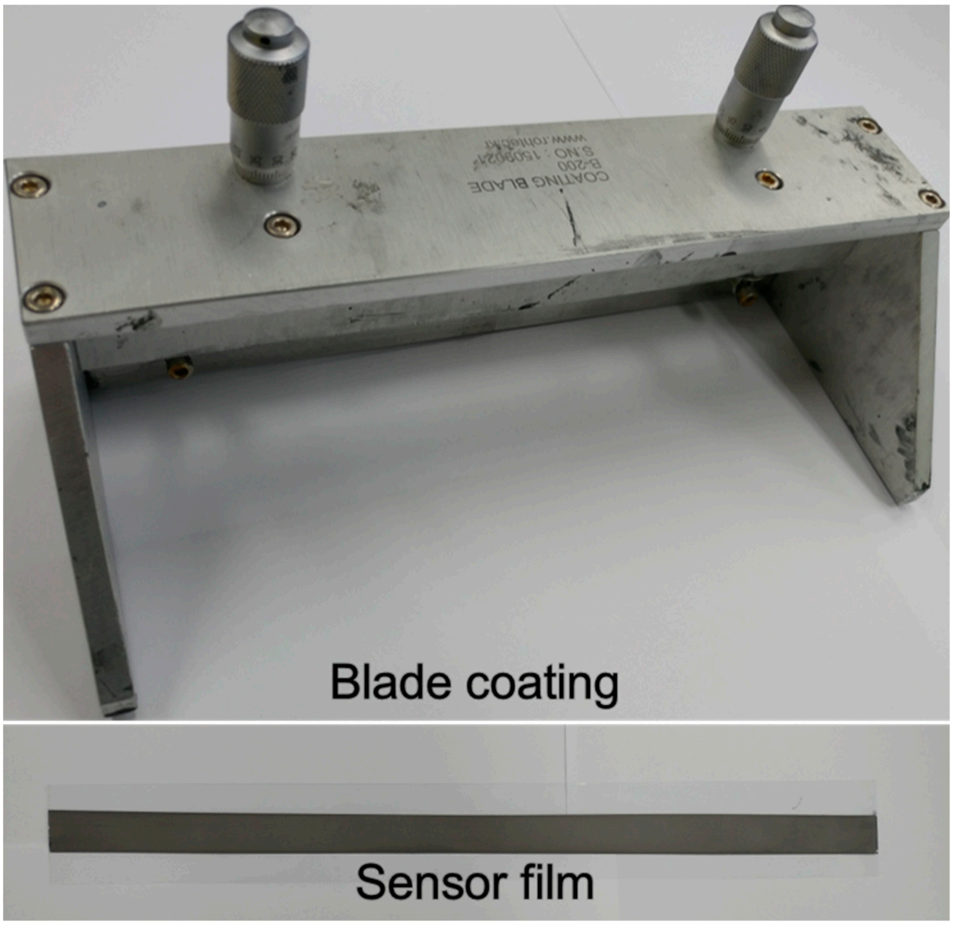

Figure S1. Photo of blade coating and sensor film.

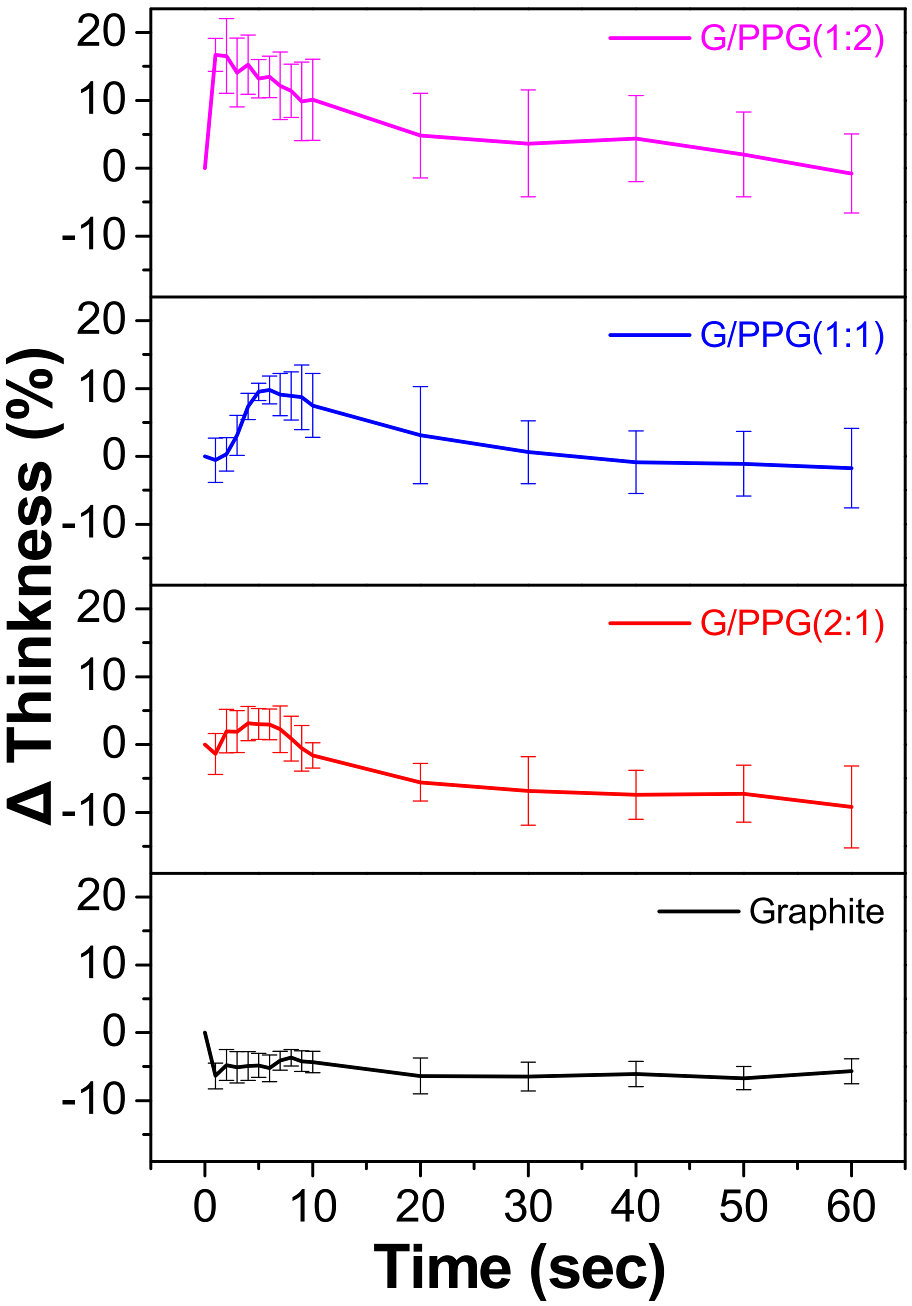

Figure S2. G/PPG HC sensor films thickness variation with error bar at dropped gasoline.

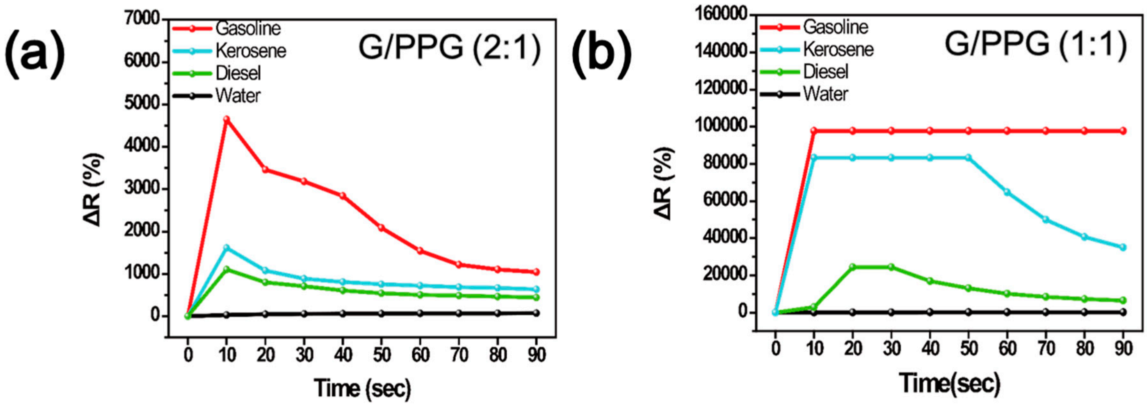

Figure S3. sensitivity of (**a**) G/PPG (2:1) and (**b**) G/PPG (1:1) HC films at different liquid organic compounds and water.

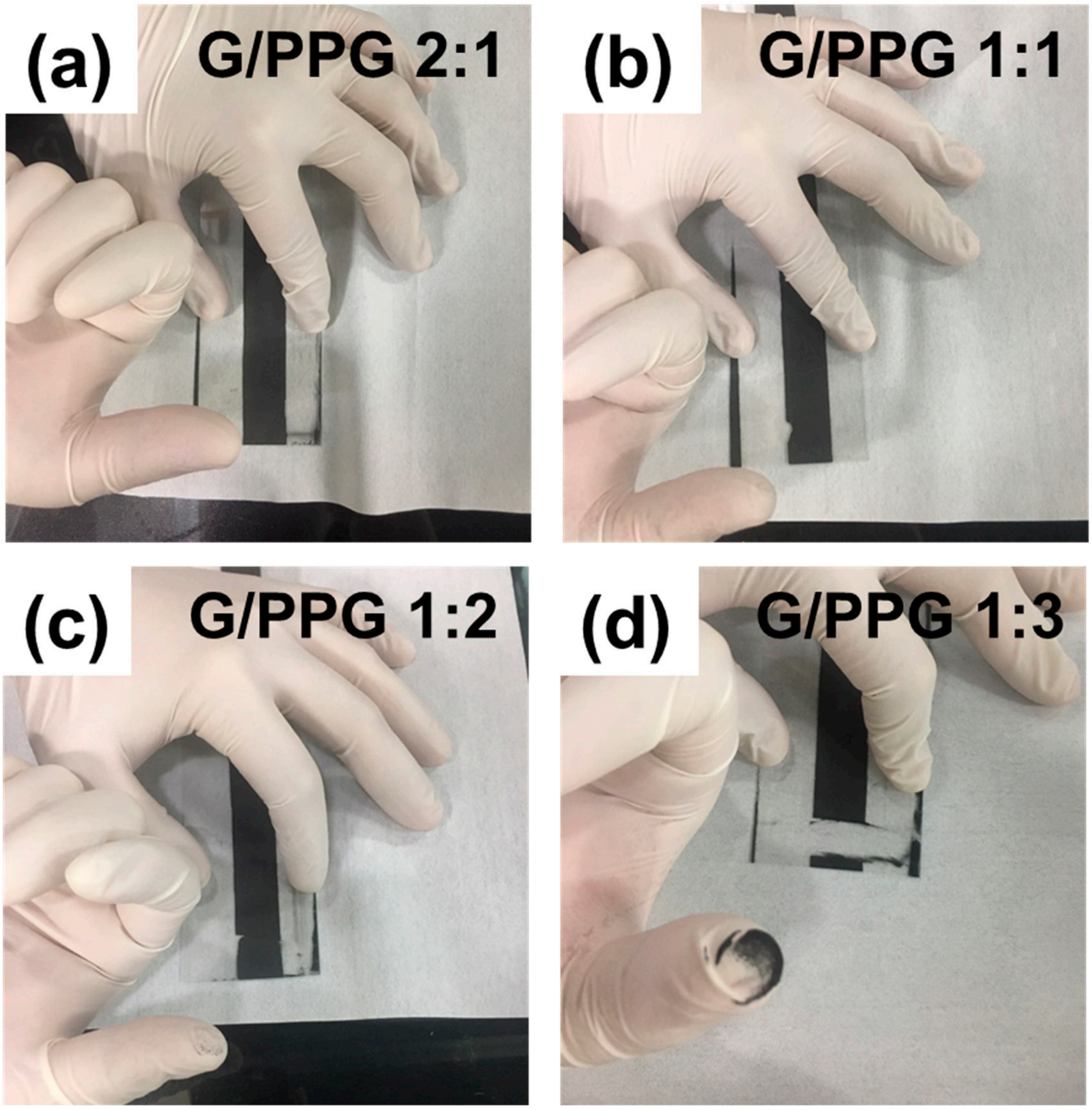

Figure S4. Photograph and mechanical stability of the G:PPG HC films on the PET film.

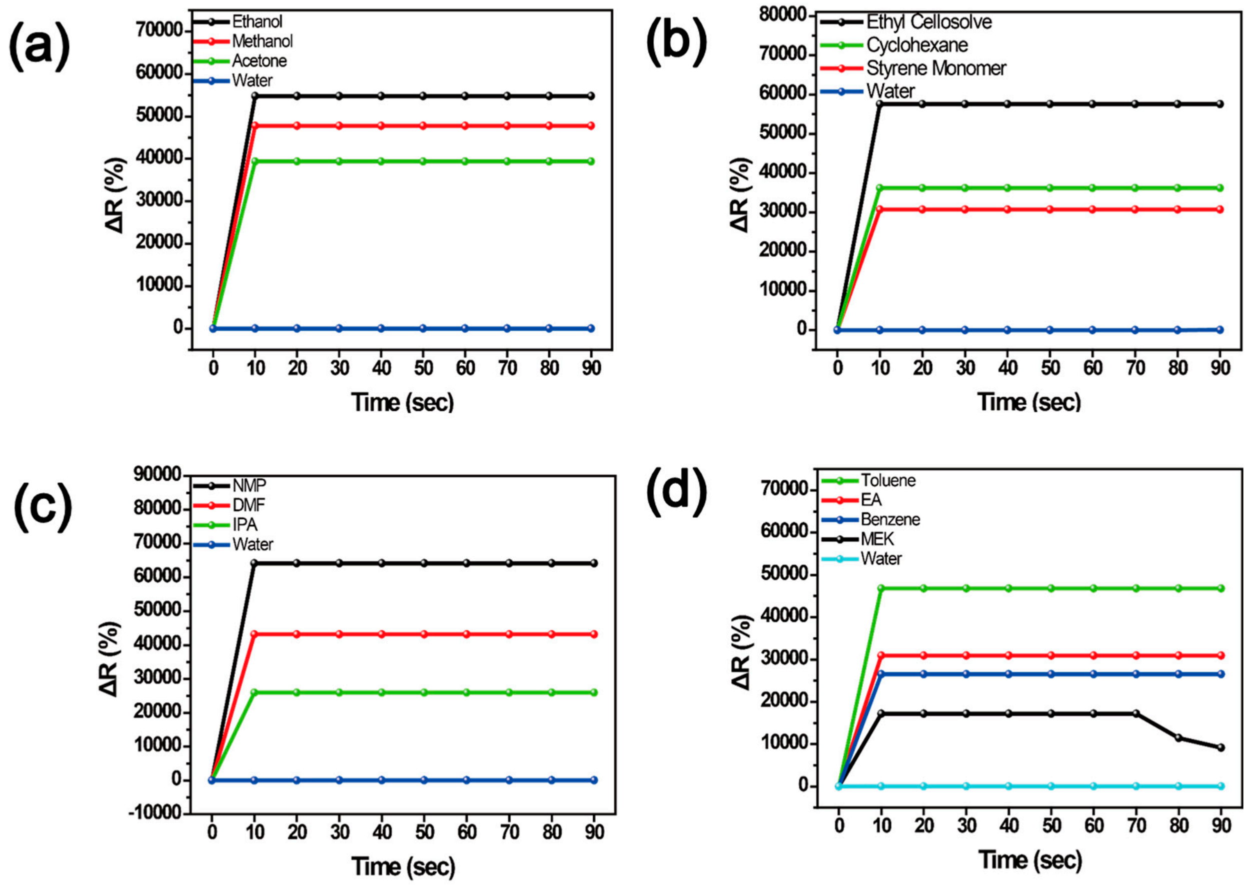

Figure S5. (**a**)–(**d**) Sensitivity of G/PPG (1:2) HC film with various liquid organic compounds.

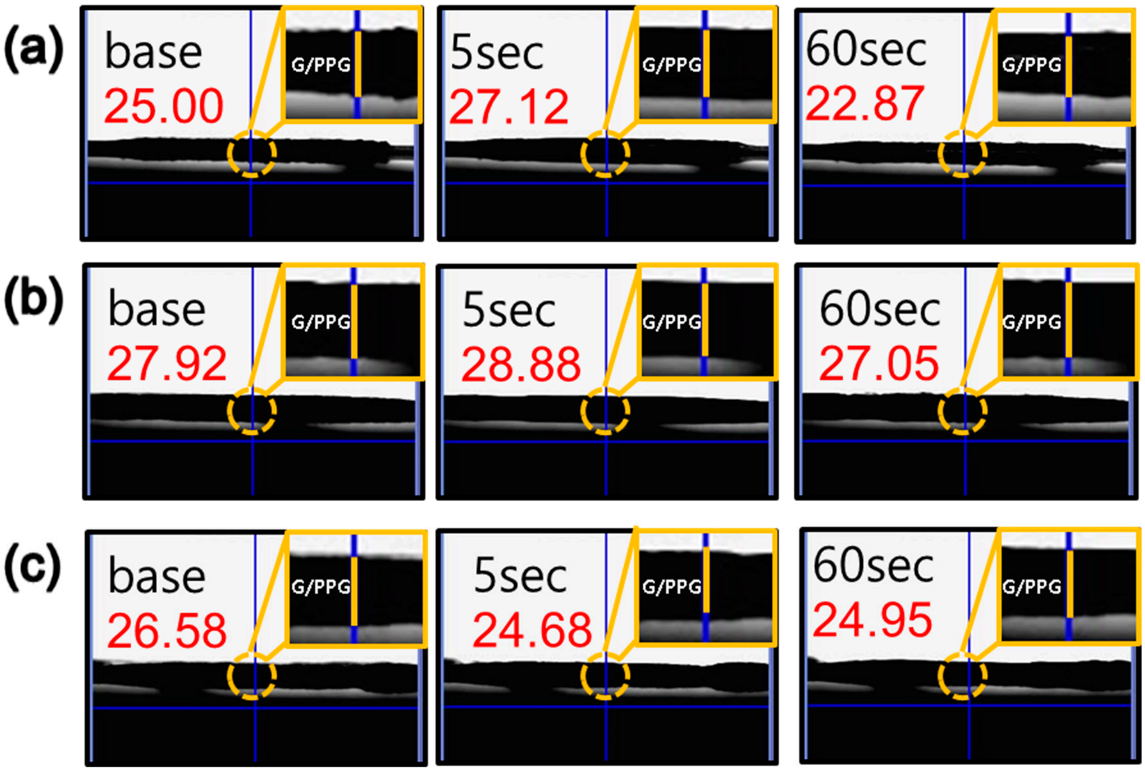

Figure S6. Swelling phenomenon of (**a**) G/PPG (1:1) HC, (**b**) G/PPG (2:1) HC and (**c**) Graphite film with gasoline.

Table S1. Zeta potential analysis of pure Graphite, G/PPG (2:1), G/PPG (1:1), and G/PPG (2:1) HC pastes.

Material	Zeta potential (mV)	Deviation
Graphite	1166.24 mV	± 46.01 mV
G/PPG (2:1)	982 mV	± 64.42 mV
G/PPG (1:1)	926.52 mV	± 70.01 mV
G/PPG (1:2)	1237.82 mV	± 43.5 mV
