# Supplementary material for: Flexible Graphite/PPG Hybrid Composite-Based Resistive Sensor for Sensing Organic Compounds
Source: Sensors (Basel). 2020 May 6;20(9):2651. doi: 10.3390/s20092651 (PMC7249171; doi:10.3390/s20092651)
Supplement: Supplementary file 1 [file sensors-20-02651-s001.zip › sensors-761390-supplementary2.docx]

Supplementary Information for:

Flexible Graphite/PPG Hybrid Composite-Based Resistive Sensor for Sensing Organic Compounds

Do Hun Kim ^1,†^, Yang Soo Lee ^2,†^, Won Kyu Park ^3,†^, Jin Sun Yoo ^1^, Changup Shim ^4^, Young Joon Hong ^5^, Bong Kyun Kang ^1^, Dae Ho Yoon ^2,^* and Woo Seok Yang ^1,^*

^1^ Nano Materials and Components Research Center, Korea Electronics Technology Institute, 25, Saenari-ro, Bundang-gu, Seongnam-si, Gyeonggi-do 13509, Korea; amazingcom@keti.re.kr (D.H.K.); yjs0415@keti.re.kr (J.S.Y.); [kangbk84@keti.re.kr](mailto:kangbk84@keti.re.kr) (B.K.K)

^2^ School of Advanced Materials Science and Engineering, Sungkyunkwan University, 2066, Seobu-ro, Jangan-gu, Suwon-si, Gyeonggi-do 16419, Korea; koami@skku.edu

^3^ Nano Material Division, Cheorwon Plasma Research Institute, Cheorwon, Gangwon-do 24047, Korea; wkpark@cpri.re.kr

^4^ Division of Advanced Materials Engineering, Kongju National University, 1223-24, Cheonan-daero, Seobuk-gu, Cheonan-si, Chungcheongnam-do 31080, Korea; [uhbi@naver.com](mailto:uhbi@naver.com)

^5^ Department of Nanotechnology and Advanced Materials Engineering, Sejong University, Seoul 05006, Korea; [yjhong@sejong.ac.kr](mailto:yjhong@sejong.ac.kr)

***** Correspondence: dhyoon@skku.edu (D.H.Y.); wsyang@keti.re.kr (W.S.Y.); Tel.: +82-31-290-7361 (D.H.Y.); +82-31-789-7256 (W.S.Y.)

† D.H. Kim, Y.S. Lee, and W.K. Park contributed equally to this work.

Received: 17 March 2020; Accepted: 29 April 2020; Published: date

**Video S1.** Video of measure of the resistance of the fabricated G/PPG sensor with non-woven fabric.

**Video S2.** Video of swelling phenomenon taken by contact angle.
